# Geographic variation in urinary tract and genital cancers in Iran: a hypothesis involving exposure to solar radiation

**DOI:** 10.1186/s13104-023-06334-x

**Published:** 2023-04-27

**Authors:** Narges Khanjani, Alireza Moradabadi, Esmail Najafi, Bagher Hayati, Reza Abdollahi

**Affiliations:** 1 Department of Medical Education, Paul L. Foster School of Medicine, Health Sciences Center, Texas Tech University, El Paso, TX USA; 2 Molecular and Medicine Research Center, Khomein University of Medical Sciences, Khomein, Iran; 3 Department of Public Health, Khalkhal University of Medical Sciences, Khalkhal, Iran; 4 Department of Environmental Health, Khalkhal University of Medical Sciences, Khalkhal, Iran; 5 Department of Nursing, School of Medical Sciences, Khalkhal Faculty of Medical Sciences, Khalkhal, Iran

**Keywords:** Cancer, Urinary tract, Genital neoplasms, Latitude, UVB

## Abstract

**Objective:**

Sunlight and vitamin D intake are considered as essential elements for human health. Insufficient intake of this vitamin is one of the causes of various cancers and some other diseases. The aim of this study was to investigate the relation between bladder, prostate, cervical and ovarian cancers with solar ultraviolet exposure in Iran. In this ecological study, data from 30 provinces were studied and analyzed by correlation and linear regression tests in SPSS software version 22. Physical activity, gender, human development index, lung cancer and altitude were adjusted at population level.

**Results:**

The incidence of bladder cancer in both sexes was inversely related to ultraviolet radiation, but it was significant only in men. Unlike bladder cancer, the incidence of cervical cancer showed a positive relation with ultraviolet radiation. No relation was found between the incidence of prostate and ovarian cancers with ultraviolet radiation. Among the adjusting variables, the incidence of lung cancer (surrogate for smoking) in women had the highest coefficient in the linear regression model.

## Introduction

Cancer is one of the main causes of death in developed and less developed countries. The burden of cancer is expected to increase, partly due to increased life expectancy around the world [1, 2], and especially in less developed countries, where about 82% of the world’s population lives [2]. Urogenital cancers, especially prostate, cervix, bladder, and ovary cancer are among cancers that have a high prevalence around the world [3]. Prostate cancer was the fifth leading cause of cancer deaths worldwide in 2012 [4]. Bladder cancer is the ninth common malignancy and is the thirteenth cause of death worldwide [5]. Cervical cancer is the fourth most common cancer among women, with about 530,000 new cases happening annually and 270,000 deaths per year. Its mortality rate is 18 times higher in low-income and middle-income countries compared to high income countries [6]. Bladder cancer is the third most common cancer in Iranian men and the ninth most common cancer in Iranian women [7]. Prostate cancer has the highest incidence of cancer in Iranian men after gastric cancer [8]. In recent years, the consumption of vitamin D has been considered as an important factor for the prevention of cancer and cardiovascular disease [9, 10]. Ecological studies have shown that deaths from cancer and cardiovascular disease are higher in areas with lower levels of sunlight [10]. In addition to having a direct effect on the body, this vitamin also play a role in the metabolism of some minerals such as calcium and phosphorus and improves bone strength [11].

Many population-based studies have shown that there is an inverse relation between the UV index and some cancers such as breast, prostate, and colon cancer [12]. Also, in animal and laboratory studies this vitamin was shown to be anti-tumoral and preventative against some diseases [13, 14]. Researchers have linked the difference in cancer mortality rates in different geographical areas of the United States, with varying levels of ultraviolet radiation and vitamin D intake [15]. Laney et al. suggest that the decreasing rates of breast, prostate, and colon cancer, from the north to south is related to UV radiation [16]. Several other studies have also reported the probable preventive effects of UVB on cancer [17–19].

Iran is a country with a wide range in latitude and altitude. There is a 15-degree difference in latitude between the south and north of Iran, and this has caused various levels of solar UV radiation. According to the Iranian Ministry of Health reports, the incidence and prevalence of cancer in the northern provinces of Iran is higher than the southern regions. Najafi et al. showed that the incidence of gastric, esophagus and colon cancer, had an inverse and significant relation with solar UVB radiation in Iran [20]. Meanwhile, vitamin D deficiency is a common problem in many nations, and especially in Middle East countries. Some researchers have reported vitamin D deficiency as a pandemic [21]. Due to the high prevalence of vitamin D deficiency in Iran [22, 23] and the increased incidence of cancer in the country, further research is needed about the etiology of these cancers.

## Main text

This is an ecological study that was conducted to identify the relation between the incidence of urinary and genital cancers (bladder, ovary, prostate and cervix) with ultraviolet radiation exposure in Iran, adjusted for altitude, physical activity, gender, age, human development index, and lung cancer incidence (as a surrogate variable for smoking in the populations).

Age-standardized incidence data from 2005 to 2008, from the National Report on Cancer in Iran, Iran Ministry of Health were used. In this report, incidence was standardized for age by using the direct standardization method and using the world’s standard population [24]. The data were merged and eventually a single incidence rate was calculated. These rates were calculated as the number of cases per 100,000 people.

In this study, data about prostate, cervical, bladder, and ovarian cancer from 30 provinces were used. Ultraviolet data from the world weather site was extracted for 9 years (from June 1, 2009 to December 30, 2018). The average annual UV exposure was calculated. The altitude and latitude of cities were obtained from the Iranian Mapping Organization website.

The variable of the percent of people with low physical activity was adjusted in this study. These data were obtained from the national survey on the risk factors of non-communicable diseases [25].

Also, in order to adjust for smoking, because the data for this variable should be at least 10 years earlier than the incidence of cancer, and due to the lack of this data for the mentioned years, the incidence of lung cancer, which is highly correlated with smoking, was used. Standardized age-specific lung cancer incidence rates in both genders for the years 2004 to 2008 was used for adjusting.

Because there is a significant difference in terms of development between different provinces in Iran, and this factor may have a direct effect on the rate of diagnosis and recording of cancer, the Human Development Index (HDI) variable for 2006–2007 was also used for adjusting. This data was obtained from the Iran Statistics Center.

The normality of the data was checked by the Kolmogorov-Smirnov (KS) test. After that, because all variables were normal, parametric tests including the Pearson correlation coefficient and linear regression models were used. Before using multivariate linear regression, its preconditions, such as stability of variable variances and the non-existence of outliners, were investigated. Also, the correlation between independent variables was estimated one by one, and if two variables showed strong correlations with each other, only the main variable was entered into the multi-variable linear regression analysis. In this study, data was collected and analyzed separately for men and women. SPSS software version 22 was used for data analysis.

## Results

The findings showed that there was a negative correlation between male bladder cancer incidence and ultraviolet radiation in Iran (r = -0.45, p-value = 0.02). This negative correlation was seen in women as well, but it was not significant. Meanwhile, ultraviolet radiation showed a positive correlation with cervical cancer (r = 0.37, p-value = 0.03).

There was no correlation between the incidence of ovarian and prostate cancers during these years with solar ultraviolet radiation. In this study, altitude did not correlate with the incidence of cancers. The rate of human development index was directly correlated with the incidence of prostate cancer (r = 40, p-value = 0.02), but this index was not related to the incidence of other cancers.

According to the findings of this study (Table 1), physical activity was not related to the incidence of cancer. But, lung cancer, as a surrogate for smoking showed a positive and significant correlation with the incidence of ovarian, cervical, and bladder cancers (in both genders) and the strongest relation was seen with men’s bladder cancer (r = 0.48, p-value = 0.007).

**Table 1:** Pearson correlations between the incidence of ovarian, cervical, prostate, and bladder cancers with independent variables

Cancer		Latitude	Altitude	UV	HDI***	Lung cancer	Physical activity
*Female*							
Ovary	r* p**	-0.01 0.93	0.29 0.11	-0.07 0.68	0.16 0.39	0.39 0.02	0.01 0.93
Cervix	r p	0.29 0.09	0.07 0.69	**0.37** **0.03**	0.13 0.49	0.40 0.02	0.20 0.20
Bladder	r p	0.04 0.83	0.16 0.38	-0.16 0.39	0.001 0.99	0.35 0.04	-0.07 0.70
*Male*							
Prostate	r p	0.15 0.40	0.28 0.13	-0.17 0.36	0.40 0.02	0.22 0.23	0.004 0.98
Bladder	r p	0.25 0.13	0.21 0.24	**-0.45** **0.02**	0.15 0.42	0.48 0.007	− 0.17 0.37

*:Pearson coefficient, **: p-value, ***: human development index

**Fig. 1:**
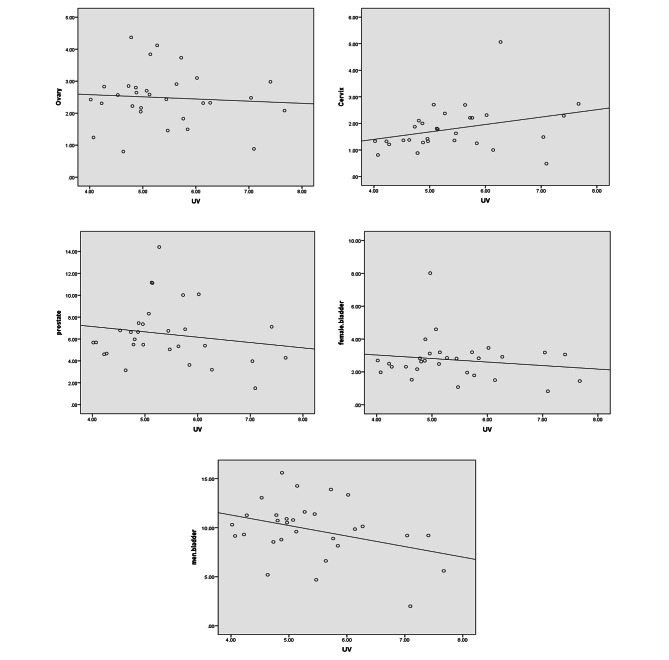
The association between exposure to solar ultraviolet radiation and the incidence of four cancers (bladder, ovary, prostate and cervix)

The association between exposure to solar ultraviolet radiation and the incidence of four cancers have been plotted in Fig. 1.

multivariate linear regression analysis, latitude was not entered into the model, because of its high correlation with UV radiation. The findings of this analysis are presented in Table 2. According to these findings, only cervical cancer showed a significant positive correlation (β = 0.45, p-value = 0.03) with UV radiation. Although male bladder cancer showed a strong significant correlation with UV in univariate models, a significant relation was not seen in multivariate linear regression. Altitude and physical activity, were not related with cancer, but lung cancer (surrogate for smoking) was directly related with the incidence of male bladder cancer and cervical cancer. The human development index also showed a direct and significant relation with prostate cancer (β = 0.36, P-value = 0.04(.

**Table 2:** Results of multivariate linear regression analysis between the incidence of ovarian, cervical, prostate, and bladder cancers with independent variables including UV

Cancer		Altitude	UV	HDI	Lung cancer	Physical activity
*Female*						
Ovary	Regression coefficient p-value	0.001 0.31	0.03 0.85	0.002 0.98	0.25 0.16	0.002 0.92
Cervix	Regression coefficient p-value	0.001 0.59	0.45 0.03	0.007 0.87	0.35 0.04	0.20 0.20
Bladder	Regression coefficient p-value	0.001 0.87	-0.12 0.62	-0.04 0.50	0.45 0.11	-0.07 0.70
*Male*						
Prostate	Regression coefficient p-value	0.001 0.42	-0.02 0.96	0.36 0.04	0.21 0.40	-0.03 0.62
Bladder	Regression coefficient p-value	0.001 0.67	-0.46 0.50	0.09 0.52	0.62 0.02	-0.37 0.53

## Discussion

In the present study, there was a negative correlation between the incidence of bladder cancer in both genders and UV radiation, but this correlation was significant only in men. A study done by Mohr et al. found similar results; and despite adjusting for smoking, UVB was a significant protective factor for bladder cancer (β = -0.332 and P-value = 0.02) [26]. In this study, although there was a moderate correlation between UVB and the incidence of bladder cancer, but multivariate linear regression showed no significant relation between these two variables. In this study, lung cancer was directly associated with the incidence of male bladder cancer. The findings of Grant also show that there is a significant inverse relation between the incidences of bladder cancer in both sexes and UV radiation [27, 28], but in the present study, the relation was not significant for the incidences of bladder cancer in women. The reason for this finding might be because of the type of Iranian women’s clothing, in which only the face and hands is exposed to the sun. This may cause insufficient vitamin D among women all over the country.

UVB-exposed skin produces vitamin D3, which researchers think reduces the incidence of some cancers. The hormonal form of vitamin D3, calcitriol, boosts the immune system by increasing the number of killer T-cells that destroy cancer cells [29]. Another study by Grant and Grant about the incidence of cancer in northern European countries suggests that 14 types of cancers, including bladder cancer, have a significant relation with UVB radiation [27].

In the Garland et al. study, all four cancers (prostate, cervix, bladder, and ovaries) were significantly correlated with UVB, but despite all other cancers that showed a negative correlation, prostate cancer showed a positive relation [30]. Godar et al. and the current study, showed that the incidence of cervical cancer increases with increased UV radiation [29]. The reason for the positive correlation between ultraviolet radiation and cervical cancer might be that ultraviolet radiation can activate some dormant viruses, including the Herpes Simplex (HSV) type 1, [31] Human immunodeficiency virus (HIV) [32] and the human papilloma virus (HPV) [29]. The human papillomavirus type 16 and 18 is the most important biological factor in the development of cervical cancer [33].

The findings of this study indicate that the incidence of only prostate cancer was related to human development index (HDI). This suggests that the incidence of prostate cancer depends on regional development. This is probably because prostate cancer occurs in older ages, and in developed areas, where life expectancy is higher than undeveloped areas.

Although no relation between UV and prostate cancer was seen in this study, Grabiec et al. showed that the incidence of prostate cancer was inversely related to the amount of ultraviolet radiation in the Netherlands [34]. This result was also seen in other [10, 35] studies. The reason that this study did not find a relation between prostate cancer incidence and ultraviolet radiation may be the incomplete registry of this cancer in the country, and the different developmental levels among the counties, which can cause incomplete registration or not diagnosing this disease. The investigations of the Iran Ministry of Health show that in the years 2004, 2005, 2006 and 2007, respectively, the coverage of the country’s cancer registration report was not complete and was about more than 70%, 81%, 83% and 86.7% of all cancer cases respectively [24].

Ovarian cancer did not show a significant relation with latitude and ultraviolet radiation in this study. But Walentowicz et al. found that the incidence of ovarian cancer was higher in the northern countries, indicating that there is a probable relation between latitude or maybe sunlight with ovarian cancer [36]. Using data from 175 countries, Garland et al. also found that the incidence of ovarian cancer significantly correlates with solar UVB (β = -0.22, p = 0.002) and is a protective factor against this cancer [30]. The reason for the lack of correlation between ovarian cancer and UV radiation in this study may be related to Iranian women’s cover (hijab) that makes UV exposure similar throughout the country. The other reason may be incomplete registration of cases in this country.

The issue that should be explained is that In this study, the variable of UVB radiation has been used for the years 2009 to 2018, because the increase of UVB radiation entering the earth is due to the destruction of the ozone layer. This happened only in Antarctica in a significant way, but other regions did not have noticeable changes. As a result, the amount of UVB received in one year can be considered similar to other years, but in this study, for more validity, the average of 9 years that was available was used [37].

Finally, it should be explained that despite the fact that there is strong correlation between solar ultraviolet radiation and latitude but, it is impossible to say with certainty that areas with higher latitude necessarily have less ultraviolet radiation. This is because ultraviolet radiation does not depend solely on latitude, and factors such as altitude, cloud coverage, and surface reflection are also involved. For example, the average UV index in Fariman in Esfahan province with a latitude of 35.7°N degrees is equal to 5, while the UV index in the city of Bardsir, in Kerman province with a latitude of 29.92°N is 5 as well. Meanwhile, Faryab in Kerman province has a latitude close to Bardsir (28.05°N), but its average UV index is 7.75.

### Limitations

This was an ecological study based on population data, and like any other ecological study may suffer from ecological fallacy. We were not able to control for many confounders including the use of sunscreen lotions which is more prevalent in woman, or the hours of working outdoors in sunlight which is usually higher in men.

## Data Availability

Data may be inquired from the corresponding author upon reasonable request.
